# Stiff-Person Syndrome: A Case Report and Treatment Modalities

**DOI:** 10.7759/cureus.94051

**Published:** 2025-10-07

**Authors:** Justin Nguyen, William B Musser, Mason Hornsby, Matthew D Overturf, Rishi Pathak

**Affiliations:** 1 Medical School, Edward Via College of Osteopathic Medicine, Monroe, USA; 2 Anatomical Sciences, Edward Via College of Osteopathic Medicine, Monroe, USA; 3 Rehabilitation, North Oaks Health Systems, Hammond, USA

**Keywords:** anti-glutamic acid decarboxylase (gad), baclofen, botox, muscle rigidity, stiff person syndrome

## Abstract

Stiff-person syndrome (SPS) is a rare autoimmune neurologic disorder characterized by fluctuating muscle rigidity and painful spasms. It is often refractory to standard therapies such as benzodiazepines, baclofen, and intravenous immunoglobulin (IVIG). We present the case of a 64-year-old African American female with SPS who experienced limited relief from opioids, muscle relaxants, IVIG, and a single intrathecal baclofen dose. Following intramuscular onabotulinumtoxinA (Botox) injections, she reported marked improvement in rigidity, posture, gait, and activities of daily living, with relief lasting approximately three months per session and no significant adverse effects. This case highlights the potential role of Botox as a safe and effective adjunctive therapy in the symptomatic management of SPS, warranting further research into its long-term efficacy and integration into treatment strategies for refractory cases.

## Introduction

Stiff-person syndrome (SPS) is a rare, progressive immune-mediated disorder of the central nervous system. It is typically characterized by diffuse, fluctuating rigidity and painful spasms of the axial and proximal limb muscles, leading to a rigid gait and frequent, uncontrolled falls [1,2]. The prevalence of SPS is estimated at 1 to 2 cases per million individuals, with females being at higher risk than males, irrespective of race [1,3]. Although the exact triggers for cases of SPS remain largely unclear, it is known to be associated with elevated titers of B-cell-mediated autoantibodies against glutamic acid decarboxylase (GAD) isoforms, which produce inhibitory neurotransmitters such as gamma-aminobutyric acid (GABA) [1-3]. Destruction of these enzyme isoforms leads to lower levels of GABA at both pre- and post-synaptic neuronal junctions, consequently causing painful muscle spasms [1].

Diagnosis and treatment of SPS are multifactorial. While there are different variants of SPS (e.g., classic, partial, progressive encephalomyelitis with rigidity and myoclonus), physicians must carefully consider clinical and symptom presentation, comorbidities, and ultimately the response to various therapies [1]. SPS is frequently underdiagnosed or misdiagnosed, and delayed recognition can result in irreversible physical disability and long-term psychological distress [1]. Although there are no definitive cures, treatment options ultimately involve symptomatic, immunomodulatory, neuromodulatory, or supportive measures [1,4,5].

We present a case involving a 64-year-old African American female diagnosed with SPS in 2015, who was referred to our Botox and migraine clinic for symptomatic management. She underwent extensive testing prior to being diagnosed, and finally found more effective relief with serial Botox injections.

## Case presentation

A 64-year-old African American female with SPS presented to the botulinum toxin and migraine clinic for evaluation of diffuse muscle rigidity. The patient reported that her symptoms began approximately 10 years ago, when she initially visited the emergency department due to an 8-9-month history of progressively worsening back pain. Over time, her condition advanced to include uncontrolled bodily movements, diffuse muscle rigidity, and spasms. Subsequently, she developed severe spasms and pain localized to the thoracolumbar region, which ultimately affected her posture and gait, resulting in a slumped-over posture with bending at the waist to the right, accompanied by an antalgic gait.

Over time, she began to develop painful muscle spasms in both her upper and lower extremities, accompanied by generalized jerking of her extremities. Despite these episodes, she consistently remained awake, alert, and oriented, with no focal motor or sensory deficits. She denied any fever, abdominal pain, constipation, diarrhea, or previous history of joint pain or swelling. While she reported that her symptoms have always been constant, they vary in severity and are often unpredictable.

The patient was initially treated with tramadol and cyclobenzaprine, which she reported did not provide any significant qualitative relief of symptoms. Other medications attempted include oxycodone, carbamazepine, diazepam, and gabapentin. She denied any history of antidepressants, tramadol, or other selective serotonin reuptake inhibitors (SSRIs) prior to her symptom onset. She does not have a history of illicit drug use, alcohol consumption, or smoking. During an emergency department visit after her breakthrough with her muscle pain, she reported relief with intravenous hydromorphone, although its effect lasted only 1-2 hours, and other analgesics were ineffective. Additional treatment modalities included intravenous immunoglobulin (IVIG), which yielded minimal improvement. According to the patient, she has received a one-time dose of intrathecal baclofen at 25 mcg, which provided eight hours of symptomatic relief.

**Figure 1:**
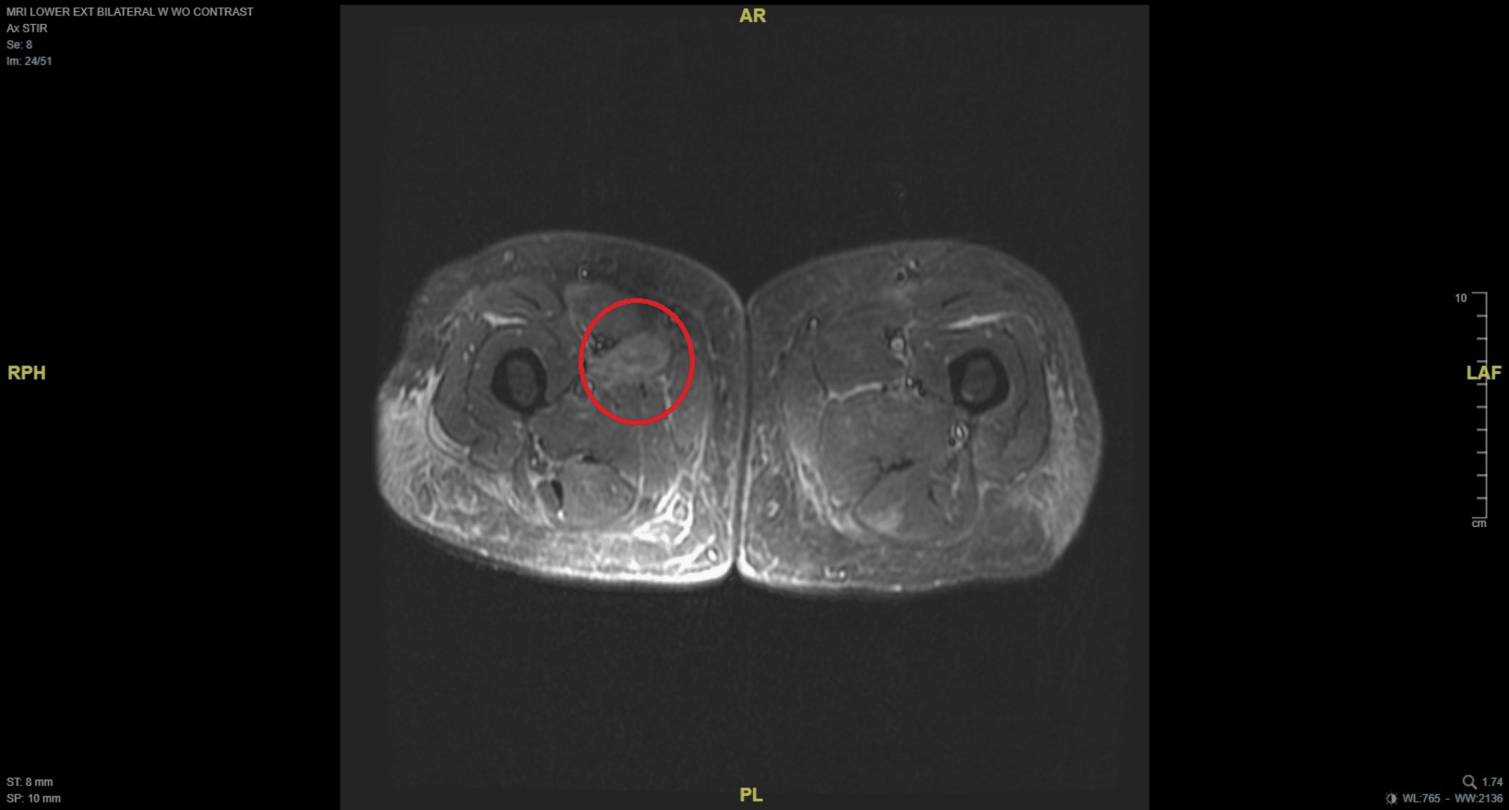
Axial MRI of both lower extremities with a red circle highlighting the area of hyperintensity in the right lower extremity.

**Figure 2:**
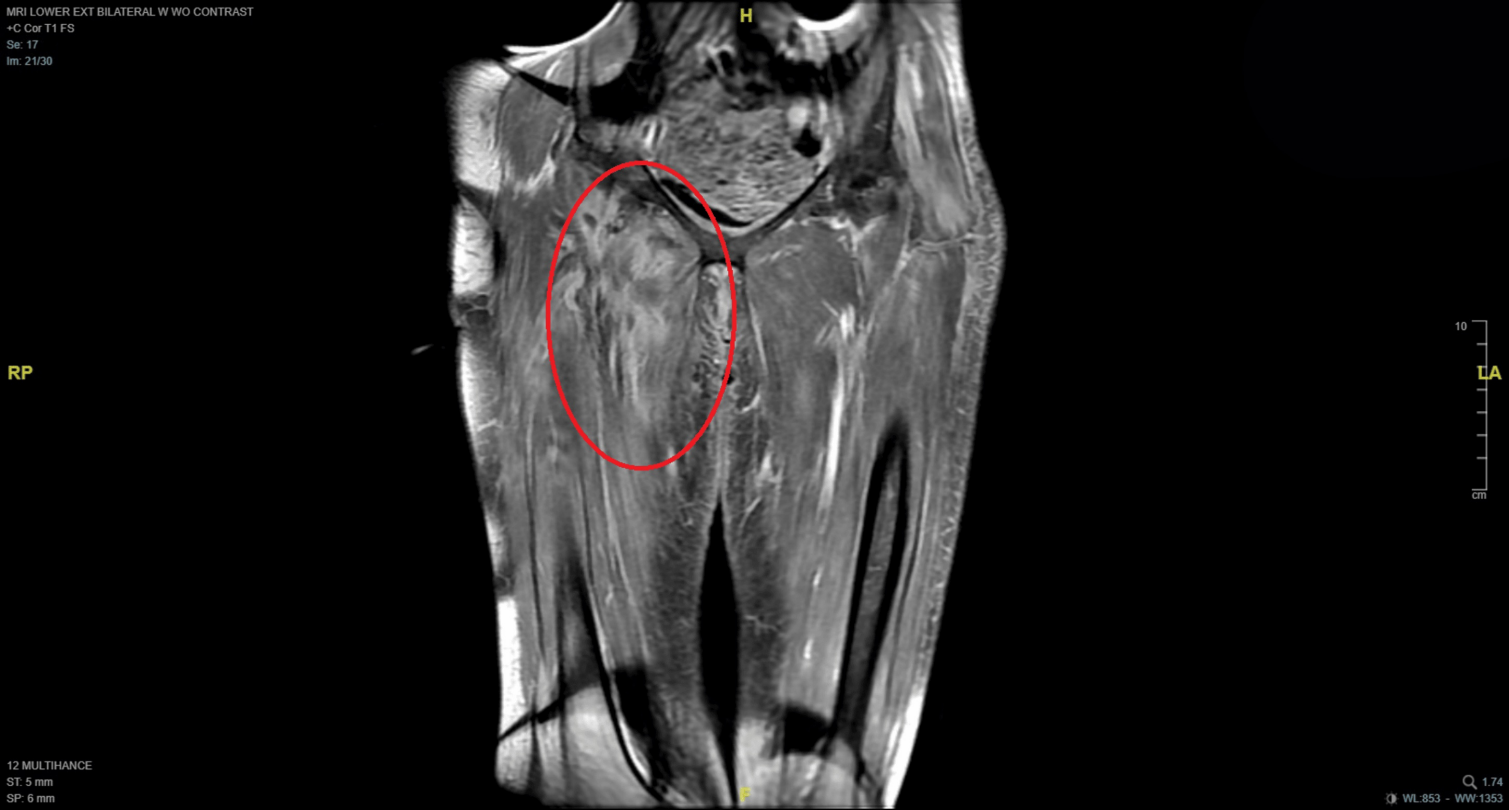
Coronal MRI of both lower extremities with a red circle highlighting the area of hyperintensity in the right lower extremity.

Her initial diagnostic workup upon presentation to the emergency department encompassed radiography, computed tomography (CT), and colonoscopy with biopsy, all of which yielded unremarkable results. Serum creatine phosphokinase (CPK) testing yielded a result of 2,009 U/L, and an MRI was obtained to evaluate for possible myopathic or inflammatory processes given the patient’s progressive, painful rigidity and muscle spasms. The MRI of the right thigh demonstrated T2 hyperintensity in the anterior medial musculature, including the adductor longus and brevis, with contrast enhancement leading to initial diagnostic consideration of possible myositis. Figures 1-2 display axial and coronal MRI views of both lower extremities. At the time of her presentation, a diagnosis of SPS was not considered, thus delaying the orders for anti-GAD antibody testing.

Initial consideration was given to inclusion body myositis based on the radiologist’s suggestions. Inclusion body myositis (IBM) was considered, given MRI findings and elevated CPK; however, the clinical presentation was inconsistent, as IBM typically manifests with progressive weakness, particularly of the quadriceps and finger flexors, rather than painful rigidity and spasms as observed in this patient. Regardless, subsequent antibody testing was obtained, and the results are illustrated in Table 1. Anti-GAD was positive (>30,000 IU/mL), confirming the diagnosis of SPS. The markedly elevated anti-GAD antibody titer alongside the clinical phenotype made SPS the more likely diagnosis, and ultimately resulted in patient refusal of the planned muscle biopsy to rule out IBM due to the invasiveness of the procedure.

**Table 1:** Autoantibody panel with elevated GAD antibody (>30); ANA, RF, and SMA negative. GAD: glutamic acid decarboxylase; RF: rheumatoid factor; ANA: antinuclear antibody; SMA: smooth muscle antibody

Pertinent Lab Test	Result	Normal Reference Value
Antinuclear Antibody (ANA)	Negative	Negative
Rheumatoid Factor (RF) Antibody	Negative	Negative
Glutamic Acid Decarboxylase (GAD) Antibody	>30,000 IU	<5 IU
Smooth Muscle Antibody (SMA)	<1:20	<1:20

**Figure 3:**
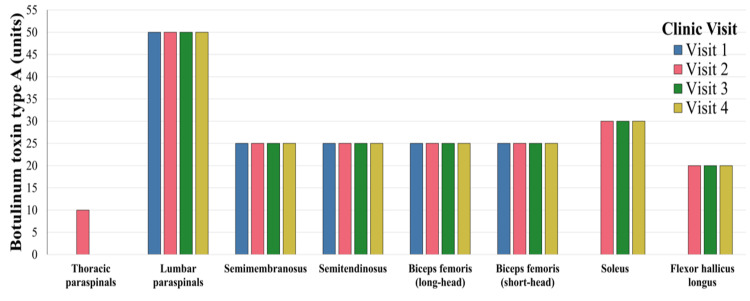
Targeted onabotulinumtoxinA dosing (units) across four clinical visits for symptomatic management. The accompanying bar graph illustrates the number of units administered to individual muscles bilaterally at each treatment session, with muscles arranged anatomically from proximal to distal. Colors represent separate clinical visits. Thoracic paraspinals were targeted only during her second visit.

Following this evaluation, the patient attended the clinic to undergo onabotulinumtoxinA (Botox) injections as part of her diagnostic process. After the initial consultation and administration of the first set of injections, she reported a significant improvement in muscle rigidity and overall functional status. Her botulinum toxin regimen and targets were selected based on the most significant patient complaints at the time of each clinic visit (Figure 3). Additionally, the patient noted considerable qualitative enhancement in her activities of daily living (ADLs). She is able to perform both upper and lower body dressing without notable difficulty. Although symptomatic relief persisted for approximately three months before subsequent treatment was necessary, she indicated that no previous therapies had yielded comparable benefits. The patient also reported no adverse effects from the botulinum toxin treatment. In subsequent visits, her Botox regimen was tailored to address specific areas of rigidity responsible for the most significant patient’s complaints at each visit. 

## Discussion

Current therapies

SPS remains a therapeutic challenge due to its rarity, unclear pathophysiologic triggers, and limited research and evidence available. GABAergic agents, including baclofen and benzodiazepines, are utilized as initial therapy for SPS [6]. IVIG, immunomodulating agents, and/or autologous hematopoietic stem cell transplantation are employed in refractory cases [6]. Long-term efficacy research remains limited. There are no formally accepted diagnostic criteria for SPS, and establishing a diagnosis requires a high index of suspicion by the physician, which often results in delayed recognition and advanced disease at presentation [1,7]. While initial therapies can afford symptomatic relief for some patients, others require extensive combination therapies, which increase the risk of developing tolerance, dependence, and potentially harmful drug interactions [7]. Despite the administration of aggressive treatments, including opioids, muscle relaxants, GABAergic agents, and intravenous immunoglobulin, medical management had previously been insufficient to adequately control the patient’s symptoms.

Role of onabotulinumtoxinA

One approach to the management of SPS that is currently under investigation involves the administration of intramuscular injections of onabotulinumtoxinA (Botox). Botox is a neuromuscular-blocking agent that operates by inhibiting the release of acetylcholine from nerve terminals at the neuromuscular junction, resulting in localized and temporary muscle paralysis [8]. Presently, this medication is predominantly employed in the treatment of spasticity and migraines [8]. Within limited cases, Botox has demonstrated potential as an adjunctive therapy for the symptomatic management of SPS. The distinctive mechanism of action of Botox may serve as a physiological ‘buffer’ for the decreased levels of GABA observed in SPS patients, by reducing the secretion of acetylcholine, the molecule responsible for muscular contraction, thus normalizing the GABA: acetylcholine ratio to its typical physiologic range [1].

Case implications

In this instance, the patient received four sessions of Botox injections administered to various muscle groups, including the paraspinal, hamstring, and lower leg muscles (Figure 3). From the inaugural session, the patient experienced significant symptomatic relief within two weeks, reporting an average of 90 days of remission prior to symptom recurrence. Although studies investigating the use of Botox injections in the management of SPS are limited, they indicate potential benefits. In a retrospective cohort of 22 seropositive SPS patients, repeated botulinum toxin injections increased patient-reported improvement from 64% to 81% and mean Likert scores from 3.78 to 4.53 (on a 1-5 scale, higher = better response) between the first and third treatments, with supramaximal doses being well-tolerated without adverse effects [9]. While evidence remains sparse, existing data suggest that periodic Botox injections in the symptomatic treatment of SPS diminish rigidity, painful spasms, and the necessity for systemic therapies, and contribute to meaningful improvements in activities of daily living [7]. Given its favorable side effect profile and broad availability, Botox constitutes a worthy therapeutic option that merits further investigation for effective SPS management. This case further supports the potential for Botox to serve as a longer-lasting treatment modality for SPS.

Future direction

Another potential option for refractory SPS is the continuous intrathecal baclofen delivery via an implanted pump [10]. The utilization of a baclofen pump administers the medication directly into the cerebrospinal fluid, thereby achieving a more effective therapeutic response at substantially lower doses compared to oral administration [11]. Intrathecal baclofen is principally indicated for patients with severe spasticity who are unable to tolerate the adverse effects or are refractory to high-dose oral medications [11]. Given that the patient discussed in this report previously exhibited a positive yet transient response to intrathecal baclofen, the prospective utility of a pump providing continuous infusion could offer the sustained benefit that other methods have failed to deliver. The underlying mechanism of intrathecal baclofen's efficacy in this condition is based on its role as a GABA B receptor agonist, which aids in restoring inhibitory tone to motor neurons, thereby mitigating rigidity and spasms characteristic of SPS [12]. While the evidence is predominantly limited to case reports, this approach suggests that continuous intrathecal baclofen administration may provide significant relief of SPS symptoms [13]. In light of these considerations, an intrathecal baclofen pump may represent a viable option for long-term management of symptoms in patients with advanced SPS refractory to conventional therapies. Following discussion of risks and benefits, the patient is deferring the intrathecal baclofen pump therapy and expressed no current interest in pursuing this option, secondary to the invasiveness of the procedure and concerns about possible complications.

## Conclusions

SPS remains a rare and intricate disorder that is managed through a diverse array of treatment modalities, which often yield only minimal symptomatic relief. Although Botox is frequently referenced in scholarly literature as an adjunct therapy in the management of SPS, its utilization is often constrained, as benzodiazepines and baclofen are recognized as the primary first-line treatments for this condition. Specifically, targeted Botox injections in the most rigid or painful muscles can offer meaningful improvement in ADLs and quality of life, particularly when symptoms are localized rather than widespread. Careful patient selection is essential, as extensive injections in multiple muscle groups may increase the risk of iatrogenic weakness and unintended spread of the toxin. Similarly, although continuous intrathecal baclofen infusion is an established therapy for refractory SPS, its use may be underrecognized, and further study could help clarify which patients might benefit most. Further research is needed to better define long-term efficacy and optimal patient selection for these therapeutic strategies in SPS and related disorders.
